# Glioma features and seizure control during long-term follow-up

**DOI:** 10.1016/j.ebr.2023.100586

**Published:** 2023-01-13

**Authors:** Leena Ollila, Reina Roivainen

**Affiliations:** Department of Neurology, Neurocenter, Epilepsia Helsinki, Helsinki University Hospital and University of Helsinki, Finland

**Keywords:** ASM, antiseizure medication, Diffuse glioma, Epilepsy, Long-term follow-up, Seizure control, Seizure freedom

## Abstract

**Background:**

An epileptic seizure is a common presenting symptom of glioma, or epilepsy may develop later during the disease. Epileptic seizures affect the quality of life in patients with glioma. Good seizure control during 6–12 months follow-up has been associated with gross total resection, radiation therapy and chemotherapy of gliomas. Little is known about seizure control during long-term follow-up and about factors which may affect the prognosis of epilepsy in glioma patients.

**Methods:**

We identified retrospectively all adult patients with diffuse glioma (grade 2–4) associated epilepsy (n = 123) living in Helsinki, who received treatment at Helsinki University Hospital neuro-oncology center during 2013–2015. We excluded patients with histopathological diagnosis prior to 2005. Data was collected from medical records for five years after diagnosis of glioma, or until death.

**Results:**

In this patient cohort 49 (39.8 %) had grade 2 glioma, 19 (15.4 %) had grade 3 glioma and 55 (44.7 %) had grade 4 glioma. 29 (23.6 %) of tumors were astrocytomas, 24 (19.5 %) were oligoastrocytomas, 15 (12.2 %) were oligodendrogliomas and 55 (44.7 %) were glioblastomas. A seizure was the presenting symptom in 87 (70.7 %) of the patients. The majority, 68 (57.6 %) patients were seizure-free for at least 12 months at some point during follow-up and 47 (39.8 %) patients were seizure-free during the last year of follow-up. Survival for five years from glioma diagnosis (p < 0.001), lower grade of tumor (p < 0.001), IDH mutation (p < 0.001), epilepsy as first symptom (p < 0.001), younger age (p < 0.001) and lack of progression (p = 0.021) correlated with seizure freedom at the end of follow-up. When the results were analyzed separately in survivors and deceased patients, only progression correlated negatively with seizure freedom at the end of follow-up in surviving patients (p = 0.008). In 5-year survivors, longer seizure-free periods were achieved by patients without progression of glioma (p = 0.040) vs patients with progression, or without focal aware (p 0.003) or focal impaired awareness seizures (p = 0.002) vs patients with only focal to bilateral tonic-clonic seizures. In deceased patients, progression (p < 0.001) and lower grade of glioma (p = 0.003) correlated positively and focal aware seizures negatively (p = 0.021) with a longer seizure-free period. In all patients, freedom of seizures at the end of follow-up was less likely for patients who had focal aware (p = 0.015) than for patients without focal aware seizures.

**Conclusion:**

There are differences in seizure-free times in patients with grade 2–4 glioma and epilepsy. The results suggest that the prognosis of glioma may be the most important factor influencing the prognosis of epilepsy.

## Highlights

• The prevalence of epilepsy in diffuse glioma patients was 75.6%.
• A seizure was the presenting symptom in 70% of glioma patients with epilepsy.
• Seizure freedom for at least one year was achieved in 57.6% of the patients.
• Bilateral convulsive seizures were the only seizure type associated with seizure freedom.
• Glioma progression was most strongly associated with short seizure-free periods.

## Introduction

For 30–50 % of patients with brain tumors, an epileptic seizure is the presenting clinical sign of a tumor; 10–30 % will develop seizures later in the course of the disease [1]. Epileptic seizures are more common in low grade gliomas (grade 1–2) than in high grade gliomas (grade 3–4). A seizure frequency of 60–85 % is seen in low-grade astrocytomas and oligodendrogliomas. In glioblastoma multiforme, the incidence of epilepsy varies from 30 to 50 % [1].

Epilepsy preceding glioma diagnosis <1 year is a favorable prognostic factor in grade 2–4 diffuse glioma [2]. Recurrence or worsening of seizures may be a sign of tumor progression [3]. According to a recent large population-based study, epilepsy may negatively impact survival in patients after glioma diagnosis and resection [4]. Epileptic seizures affect quality of life, especially in cases of refractory epilepsy [5]. Antiseizure medication (ASM) treatment is recommended after the first seizure, when a cerebral glioma is identified as etiology of epilepsy [6]. Improved seizure control has been associated with gross total resection, radiation therapy and chemotherapy of glioma during 6–12 months follow-up [7], [8], [9], [10], [11].

Less is known about seizure control during long-term follow-up. Several factors which affect the prognosis of glioma have been found [12], [13], [14], but their significance regarding the prognosis of epilepsy is less well-known.

In the present study, our aim was to describe the epilepsy outcomes in a population-based cohort of patients with glioma-related epilepsy representing different phases during the course of the disease. Analysis of patient-related factors that may associate with seizure outcomes, such as comorbidities was included [15]. We identified the prevalence of epilepsy and refractory epilepsy in this patient population and attempted to identify glioma-related and other factors contributing to seizure control during glioma treatment and long-term follow-up.

## Materials and methods

The study is a retrospective observational cohort study. It conforms to the Finnish legislation concerning medical research, and the study permission was granted by the Helsinki University Central Hospital (HUCH) Neurocenter Institutional Review Board.

### Selection of patients

We identified all cerebral glioma patients in contact with Helsinki University Hospital Neurology or Oncology Departments during 2013–2015 according to the ICD 10 diagnosis codes (C71). Patients were included, if they had a histopathological diagnosis of World Health Organization (WHO) grade 2, 3 or 4 glioma, 2) histopathologic diagnosis of glioma was made after 2004 and 3) the seizure(s) lead to the diagnosis or occurred within five years after diagnosis of glioma. A neuropathologist made the histopathological diagnosis. All glioma patients living in Helsinki are treated at Helsinki University Hospital and only patients living in Helsinki were included. The corresponding number of glioma patients alive was checked from The Finnish Cancer registry, which has high accuracy for glioma patient identifications [16]. Patients may have additional visits to occupational health care or other units, but neuro-oncological follow-up of the brain tumor is at Helsinki University Hospital. Patients were excluded, if the histopathological diagnosis was some other tumor than astrocytoma, oligodendroglioma, oligoastrocytoma or glioblastoma, if the diagnosis was pilocytic astrocytoma, or if the primary tumor was outside the brain. Patients with unavailable follow-up data due to moving from the Helsinki University Hospital catchment area were also excluded.

### Treatment of patients

Glioma associated epilepsy was diagnosed after the first seizure caused by glioma. ASM was usually recommended after the first seizure. Treatment and MRI follow-up was planned by the neuro-oncological team and clinical follow-up of epilepsy was performed by a neurologist. The changes to ASMs were carried out according to clinical protocols at the respective time. The purpose of surgical resection was nearly always the safe removal of the tumor, without an attempt to delineate or remove the epileptogenic cortex. Patients received radiation therapy and chemotherapy according to oncology protocols of the respective time.

### Data collection

Data was collected from medical records for five years from the diagnosis of glioma or until death. Included were patient gender, age at diagnosis, date of glioma diagnosis, histopathological diagnosis according to neuropathologist, presence of IDH mutation, location and first symptom of glioma, time and type of surgery and oncological treatments and time of tumor progression. Epilepsy data consisted of date of first seizure, previous epilepsy, seizure types, status epilepticus, ASM treatment and seizure-free periods. Seizure outcome was assessed by 1) length of longest seizure-free period, 2) whether a 12-month remission period was achieved and 3) seizure freedom at end of follow-up. Presence of other somatic disease was recorded when diagnosis had been made of any other neurological disease or other disease with need of hospital and/or laboratory value follow-up and/or drug treatment either regularly, or when required. Psychiatric disease was considered present when diagnosis had been made of neurodevelopmental, depressive, bipolar, anxiety, psychotic, obsessive–compulsive, personality, trauma and stress-related, or dissosiative disorders. Abuse of alcohol or drugs was recorded according to clinical judgement of the treating physician. Imaging analysis included conventional T1- and T2-weighted magnetic resonance images and gadolinium enhancement. Neuroradiologist evaluated the progression on MRI. Histopathological classification was determined using the classification of brain tumors approved by the World Health Organization 2000 or 2007. Detection of IDH1 mutation in all patients with grade 2–4 glioma at the time of resection or biopsy started in 2011 with immunohistochemistry and identification of IDH 1 and 2 mutations by sequencing started in 2014. In some cases, IDH mutation was detected later retrospectively according to clinical need. In Helsinki University Hospital, the clinical protocol is to start the detection of IDH mutation with immunohistochemistry. If this is negative, sequencing is used at least for all patients with grade 2–4 glioma, excluding patients with grade 4 glioma and age over 55. Other molecular markers were analyzed according to the protocols of the respective time, but were not collected. Histopathological progression on re-resection was recorded. Seizure types were classified according to the ILAE 2017 Classification [17] focal aware, focal impaired awareness, or focal to bilateral tonic-clonic seizures. Data on the final phase of palliative/hospice care was not collected.

### Statistical analysis

All analyses were performed with SPSS, version 27 (SPSS Inc., Chicago, IL, USA). The association between clinicopathologic variables and freedom of seizures at the end of follow-up was examined using cross tabulation test, chi-square test, Fisher exact test and Phi and Cramer’s V. The association between clinicopathologic variables and longest seizure free time was examined using linear regression analysis. Survival analyses were performed using Kaplan-Meier model. The association between clinicopathologic variables and seizure types was examined using cross tabulation test, chi-square test, Fisher exact test and Phi and Cramer’s V. Mann-Whitney *U* test, cross tabulation test, chi-square test and Fisher exact test were used in other analysis. Values with p < 0.05 were considered statistically significant.

## Results

### Patient population

We identified 248 adult (≥18 year old) patients living in Helsinki who had visited the neurology or oncology departments during 2013 to 2015 receiving the ICD 10 diagnosis code C71 for glioma. Patients were excluded due to radiological diagnosis only, or uncertain histological diagnosis (n = 7), other tumor than diffuse astrocytic or oligodendroglial tumor such as PNET, pilocytic grade 1 astrocytoma, pineal tumor, germinoma, ependymoma, or medulloblastoma (n = 11). Primary resection had been made outside Finland or Helsinki in nine patients.

In the remaining 221 patients with diffuse gliomas, 54 patients did not have epilepsy. Eight patients had used ASMs indications including prophylactic medication [1], psychiatric disease [2], migraine [1], and temporary use due to suspected seizure episode not receiving diagnosis of epilepsy [4].

Out of the 167 patients (75.6 %) with diagnosis of epilepsy, patients were further excluded from the study cohort due to inaccuracy of the diagnosis of glioma (n = 3). Four patients developed seizures over five years from diagnosis, and 32 had received diagnosis of glioma prior to 2005. Three patients lived abroad for long periods or had late neurological follow up. Two patients had epilepsy not related to the glioma. Finally, 123 patients were included in the study.

During the follow-up year 2018 there were 165 patients with diagnosis of glioma (C71) visiting the Helsinki University Hospital neurological or oncological clinics. 122 of these patients had a diagnosis of epilepsy. 119 patients had received the diagnosis of glioma during the previous ten years, 90 during the previous five years and 30 patients during 2018. These numbers corresponded closely to the Finnish Cancer registry data in 2018, showing 122 adult patients with glioma diagnosis within last ten years, 88 during the last five years and 36 during 2018.

Data on seizure freedom at the end of follow-up was missing in five patients due to transfer to institutional care shortly after diagnosis, not committing to follow-up, alcohol abuse, drug abuse, or severe aphasia. Therefore, 118 patients were included in the final analysis of the seizure freedom at the end of follow-up. Data were missing due to similar reasons in the longest seizure-free time analysis in seven patients. Data on 12 months of seizure freedom at some point during follow-up was missing in five patients. In linear regression analysis, there were insufficient data for inclusion in eight patients. Data on longest seizure-free time was missing in seven patients and data on seizure types was missing in one patient.

### Patient characteristics

Characteristics of the patient population are shown in Table 1. A seizure was the presenting symptom of the glioma in 87 (70.7 %) of the patients. If the first symptom was other than seizure, mean time from glioma diagnosis to first seizure was 6.9 months (median 2.0 months, SD 11.9, range 0–56). Somatic or psychiatric comorbidity was diagnosed in approximately half of the patients. Patients with other somatic disease were older than patients without somatic disease (p = 0.001). Somatic comorbidity covered a wide variety of diseases from benign (e.g. migraine) to more severe (e.g. coronary artery disease). 40.2 % of patients without psychiatric disease and 71.4 % of patients with psychiatric disease were alive at the end of 5-year follow-up (p = 0.015). Only one of the 23 patients ≥65 years old was alive at the end of 5-year follow-up.

**Table 1:** Patient characteristics.

Variable	Number (%) or mean (SD) [range]
All	123(100)
Female	43 (35.0)
Male	80 (65.0)
Age	48.0 (16.8)[20–86]
Other disease	
Somatic	49 (39.8)
Psychiatric	21 (17.1)
Somatic and/or psychiatric	61 (49.6)
First symptom	
Epilepsy	87 (70.7)
Other	35 (28.5)
Incidental finding	1 (0.8)
Previous epilepsy	2 (1.6)
Tumor location	
Frontal	53 (43.1)
Temporal	18 (14.6)
Parietal	11 (8.9)
Occipital	3 (2.4)
Several lobes/large	38 (30.9)
Tumor grade	
2	49 (39.8)
3	19 (15.4)
4	55 (44.7)
IDH mutation	
Yes	53 (43.1)
No	62 (50.4)
Unknown	8 (6.5)
Seizure type*	
Focal aware	65 (52.8)
Focal impaired awareness	36 (29.3)
Focal to bilateral tonic-clonic	87 (70.7)
Other	0
Histopathological diagnosis	
Astrocytoma	29 (23.6)
Oligoastrocytoma	24 (19.5)
Oligodendroglioma	15 (12.2)
Glioblastoma	55 (44.7)
Change to more malignant during follow-up**	15 (12.2)
Progression during follow-up ***	75 (61.0)
Tumor treatments during follow-up	
Resection	108 (87.8)
Biopsy	19 (15.4)
Radiotherapy	71 (57.7)
Chemotherapy	85 (69.1)
Chemoradiotherapy	59 (48.0)
Death during follow-up	
Yes	67 (54.5)
No	56 (45.5)

*Several seizure types were possible in the same patient.

**Histopathological verification.

***During treatment leading to a change of treatment or after a stable post-treatment period.

Data on alcohol or drug abuse was often not documented in medical records. Data on abuse of alcohol was missing at the time of diagnosis 41.5 %, after diagnosis 46.3 % and abuse of drugs 88.6 % of patient files. Abuse of alcohol was identified in 8.1 % of all patients at diagnosis, but in only 1.6 % of patients after diagnosis. Abuse of drugs was identified in 2.4 % of patients.

### Seizure type

Seizure types were quite similar in different patient groups regardless of the location, extent or malignancy of the glioma (Table 2). Likewise, in patients surviving ≥5 years, seizure types were not statistically significantly different from patients who died during 5 years from diagnosis. The most common type of seizure was a focal to bilateral tonic-clonic seizure. The occurrence of focal aware seizure correlated negatively with seizure freedom at end of follow-up.

**Table 2:** Seizure types during follow-up.

Seizure type	Factor	Patient number (%)	p-value	Effect size: Cramer's V
Focal aware	Location		0.736	0.076
	Frontal	26 (50.0)		
	Temporal/parietal/occipital	19 (59.4)		
	Several/large	20 (54.1)		
	Grade		0.755	0.068
	2	28 (57.1)		
	3	9 (47.4)		
	4	28 (52.8)		
	IDH mutation		0.709	0.087
	Yes	29 (54.7)		
	No	33 (55.0)		
	Unknown	3 (37.5)		
	Seizure-free at the end of follow-up		0.015	0.230
	Yes	19 (40.4)		
	No	44 (63.8)		
	All	65 (52.8)		
Focal impaired awareness	Location		0.510	0.110
	Frontal	15 (28.8)		
	Temporal/parietal/occipital	7 (21.9)		
	Several/large	13 (35.1)		
	Grade		0.831	0.053
	2	13(26.5)		
	3	6 (31.6)		
	4	17 (31.5)		
	IDH mutation		0.377	0.123
	Yes	14 (26.4)		
	No	18 (29.5)		
	Unknown	4 (50.0)		
	Seizure-free at the end of follow-up		0.063	0.179
	Yes	9 (19.1)		
	No	25 (35.7)		
	All	36 (29.3)		
Focal to bilateral tonic-clonic	Location		0.734	0.070
	Frontal	39 (75.0)		
	Temporal/parietal/occipital	23 (71.9)		
	Several/large	25 (67.6)		
	Grade		0.250	0.146
	2	39 (79.6)		
	3	12 (63.2)		
	4	36 (67.9)		
	IDH mutation		0.772	0.079
	Yes	40 (75.5)		
	No	41 (68.3)		
	Unknown	6 (75.0)		
	Seizure-free at the end of follow-up		0.677	0.041
	Yes	33 (70.2)		
	No	51 (73.9)		
	All	87 (70.7)		

### Seizure freedom

At some point during the five years from glioma diagnosis 68 (57.6 %) patients were seizure-free for at least 12 months. One patient achieved two separate periods of 12 months seizure freedom. In 28 (41.2 %) patients with seizure recurrence, association with glioma progression was found in 17 patients (60.7 %).

Seizure freedom at the end of follow-up is shown in Table 3. Out of the 64 patients who died within five years from diagnosis of glioma, 15.6 % had been seizure-free during the last year, whereas 68.5 % of patients with survival ≥5 years after diagnosis of glioma were seizure-free. The presence vs lack of IDH mutation, seizure vs other sign as first symptom of glioma, grade 2 vs grade 4 glioma, lack of progression, and younger age correlated with a better outcome. Most of the differences were small or disappeared when patients were divided into groups based on survival. In surviving patients, only progression correlated negatively with achieving seizure freedom (Table 4). In patients surviving ≤5 years (n = 64) none of the factors reached statistical significance.

**Table 3:** Patient/glioma features and freedom of seizures at the end of follow-up.

	Seizure-free ≥1 year at the end of the follow-up (%)	Seizure-free <1 year at the end of the follow-up (%)	Total patient number	p-Value	Effect size (Phi/ Cramer's V)
All	47 (39.8)	71 (60.2)	118		
1st symptom				<0.001	−0.327
Epilepsy	42 (50.0)	42 (50.0)	84		
Other	5 (14.7)	29 (85.3)	34		
Progression during follow-up				0.021	0.222
Yes	22 (31.0)	49 (69.0)	71		
No	25 (53.2)	22 (46.8)	47		
IDH mutation				<0.001	0.420
Yes	29 (58.9)	21 (42.0)	50		
No	12 (20.0)	48 (80.0)	60		
Unknown	6 (75.0)	2 (25.0)	8		
Glioma grade				<0.001	0.374
2	29 (60.4)	19 (39.6)	48		
3	7 (41.2)	10 (58.8)	17		
4	11 (20.8)	42 (79.2)	53		
Location				0.206	0.165
Frontal	25 (49.0)	26 (51.0)	51		
Temporal/parietal/occipital	10 (31.3)	22 (68.8)	32		
Several/large	12 (34.3)	23 (65.7)	35		
Other somatic disease				0.081	0.175
Yes	13 (28.9)	32 (71.1)	45		
No	34 (46.6)	39 (53.4)	73		
Age				<0.001	0.339
≤55	42 (50.6)	41 (49.4)	83		
>55	5 (14.3)	30 (85.7)	35		
Death during follow-up				<0.001	0.538
Yes	10 (15.6)	54 (84.4)	64		
No	37 (68.5)	17 (31.5)	54

**Table 4:** Patient and glioma features and freedom of seizures at the end of follow-up in surviving patients.

	Seizure-free ≥1 year at the end of the follow-up (%)	Seizure-free <1 year at the end of the follow-up (%)	Total patient number	p-Value	Effect size (Phi/ Cramer's V)
1st symptom				0.071	−0.268
Epilepsy	35 (72.9)	13 (27.1)	48		
Other	2 (33.3)	4 (66.7)	6		
Progression during follow-up				0.008	0.384
Yes	13 (50.0)	13 (50.0)	26		
No	24 (85.7)	4 (14.3)	28		
IDH mutation				0.802	0.113
Yes	26 (66.7)	13 (33.3)	39		
No	6 (66.7)	3 (33.3)	9		
Unknown	5 (83.3)	1 (16.7)	6		
Glioma grade				0.878	0.113
2	28 (66.7)	14 (33.3)	42		
3	5 (83.3)	1 (16.7)	6		
4	4 (66.7)	2 (33.3)	6		
Location				0.121	0.277
Frontal	21 (80.8)	5 (19.2)	26		
Temporal/parietal/occipital	7 (50.0)	7 (50.0)	14		
Several/large	9 (64.3)	5 (35.7)	14		
Other somatic disease				0.516	0.114
Yes	9 (60.0)	6 (40.0)	15		
No	28 (71.8)	11 (28.2)	39		
Age				0.535	0.078
≤55	36 (69.2)	16 (30.8)	52		
>55	1 (50.0)	1 (50.0)	2		

Patients who received chemoradiation were less likely to be seizure-free (24.1 %) at the end of follow-up than patients who did not receive chemoradiation (55.0 %) (p < 0.001). We found no difference in seizure freedom at the end of follow-up for any other treatments.

There were 14 patients who were seizure-free throughout 5 years follow-up. These patients had a seizure at the time of diagnosis and were subsequently seizure-free. In all of these patients, the tumor was resected (p = 0.212), they were younger than patients on average (p = 0.003), had more often grade 2 (n = 11) than grade 4 (n = 2) tumor (p = 0.012) and were more often progression free during the follow-up (p = 0.038). Ten (71.4 %) patients were male with mean age of 34.9 (SD 10.2, range 20–51). Five (35.7 %) patients had progression during 5-year follow-up. Tumor location was frontal in 5 (35.7 %) patients, temporal in 5 (35.7 %) patients, parietal in 1 (7.1 %) patient and several/large in 3 (21.4 %) patients.

In linear regression analysis, we analyzed longest seizure-free time of survivors and deceased patients in separate groups (Table 5). IDH mutation was excluded from the analysis due to the changes in data collection, the resulting uncertainty in data collection and multicollinearity.

**Table 5:** Factors contributing to the longest seizure-free time – linear regression.

Death during follow-up	Variable	Beta	p-value	R square
No				0.395
	Progression	−0.262	0.040	
	Somatic disease	−0.242	0.051	
	Grade	−0.115	0.348	
	Seizure type			
	Focal aware	−0.395	0.003	
	Focal impaired awareness	−0.394	0.002	
	Focal to bilateral tonic-clonic	−0.101	0.424	
Yes				0.311
	Progression	0.493	<0.001	
	Somatic disease	0.246	0.080	
	Grade	−0.359	0.003	
	Seizure type			
	Focal aware	−0.303	0.021	
	Focal impaired awareness	−0.061	0.594	
	Focal to bilateral tonic-clonic	−0.093	0.436	

Occurrence of focal aware seizures and focal impaired awareness seizures were negatively correlated with seizure-free time in patients who survived 5 years of follow-up. Focal aware seizures were likewise negatively correlated with seizure-free time in patients not surviving five years of follow-up. Progression showed a negative correlation with seizure-free times in surviving patients and a positive correlation in patients who died during follow-up.

### Survival

**Fig. 1:**
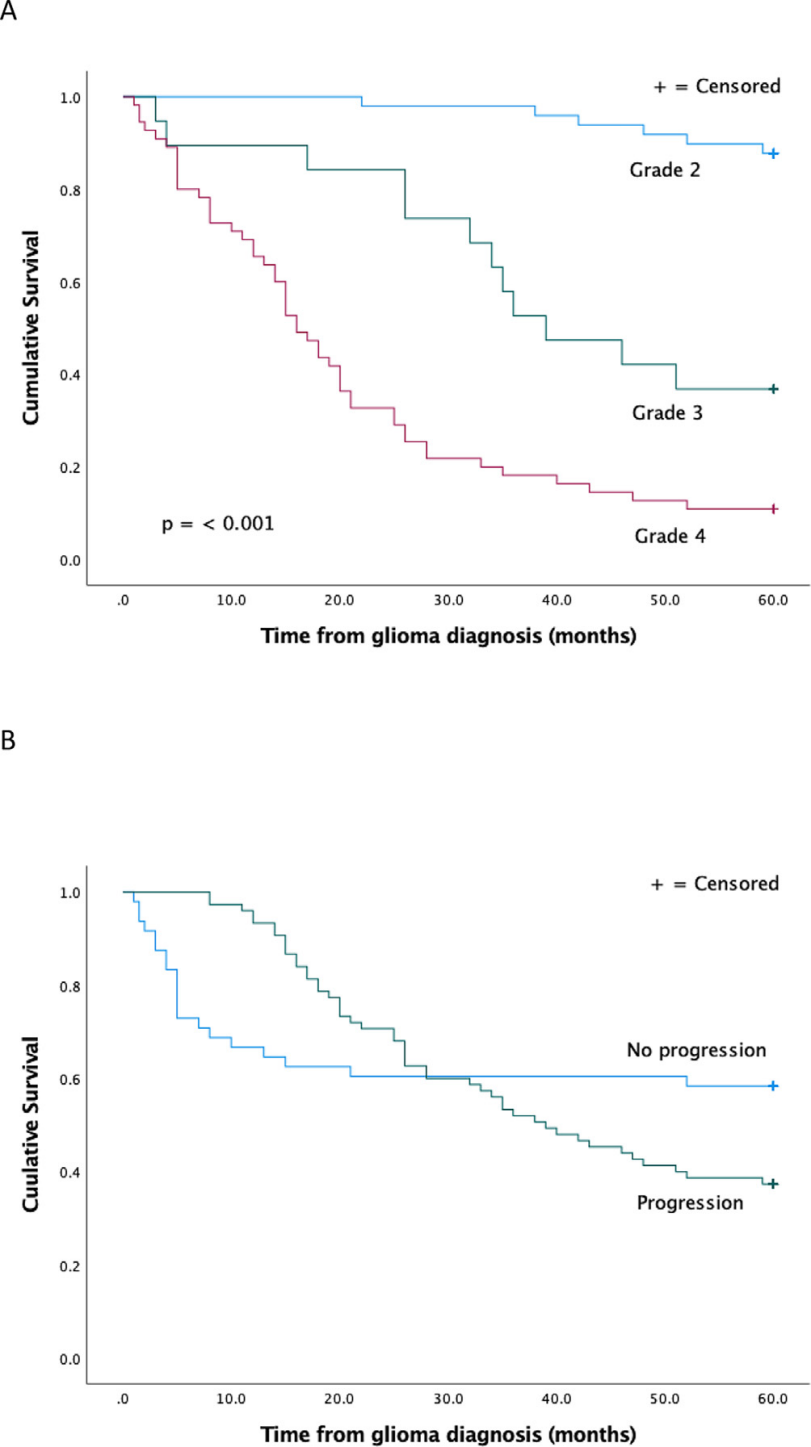

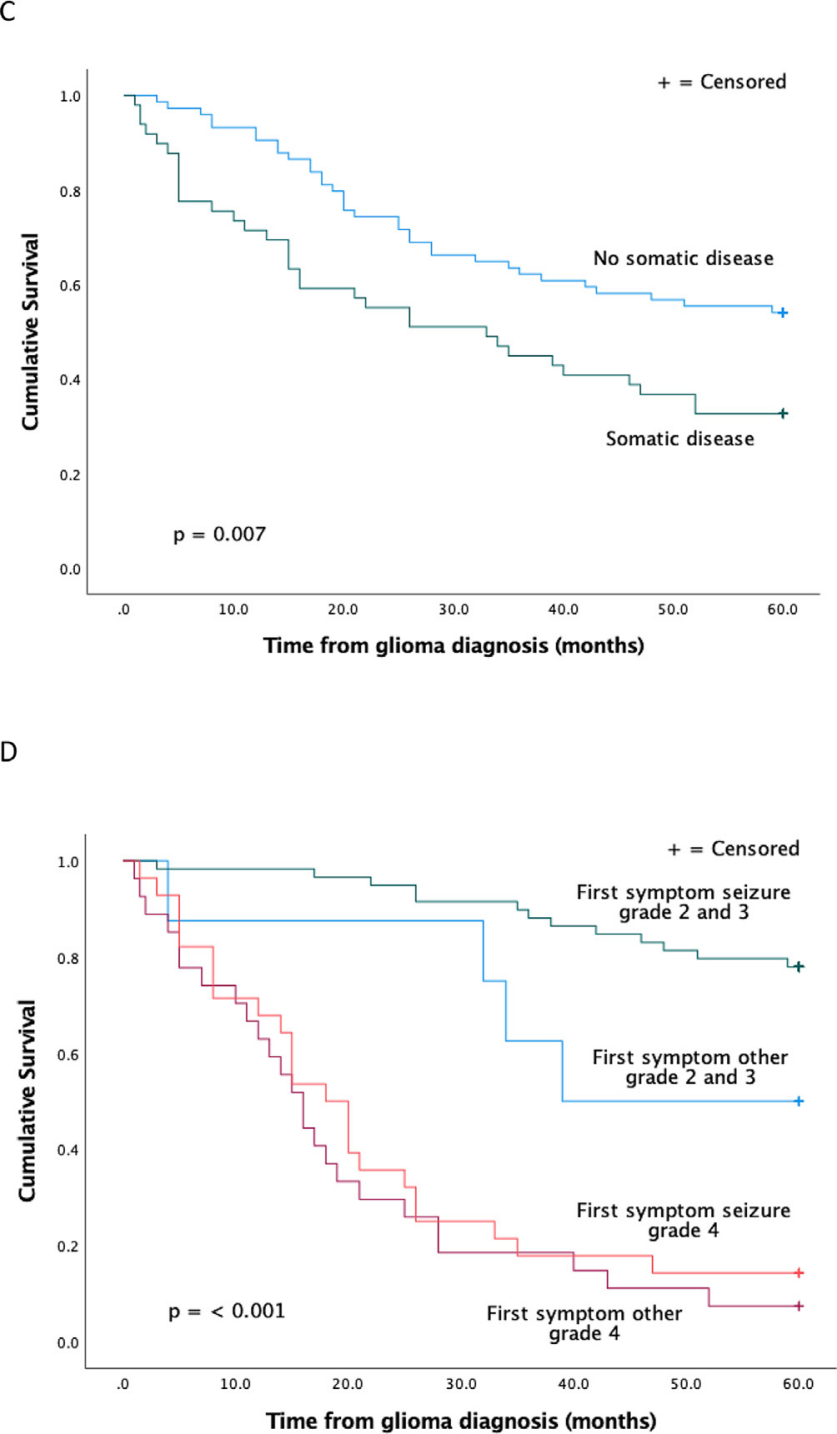
Cumulative survival by grade (**A**), in patients with or without progression, (**B**) in patients with or without other somatic disease (**C**) and by first symptom seizure vs other and grade (**D**).

In cumulative survival analysis, a higher grade of glioma correlated with shorter survival (Fig. 1). At the beginning of follow-up, progression correlated with longer survival, but later with shorter survival. Patients with aggressive glioblastomas may die shortly after diagnosis leaving no time for new progression.

Seizure as the first symptom correlated with longer survival. Because of the small size of some subgroups, grade 2 and 3 tumors were analyzed together in cumulative survival analysis by first symptom.

### ASM use and withdrawal

Nearly all patients initiated ASMs after first seizure and continued therapy throughout the study. Only one patient never received ASM treatment during the study period. After the first ASM initiation, the mean number of new ASM iniations was 1.2 (median 1, range 0–6). Levetiracetam, oxcarbazepine and valproic acid were the most commonly initiated ASMs. Other ASMs used were carbamazepine, phenytoin, lacosamide, lamotrigine, pregabalin, topiramate, clobazam and clonazepam.

ASMs were withdrawn in ten (8.1 %) patients. Four remained seizure-free, and in five patients, seizures re-appeared. The outcome is not known in one patient. In the four patients remaining seizure-free, glioma location was frontal (n = 2), or temporal (n = 2) and tumor grades were 2 (n = 2), 3 (n = 1) and 4 (n = 1). All four patients survived for 5 years after glioma diagnosis, but glioma progression was seen in one patient. In the five patients with seizure recurrence, the tumor was multilobar (n = 3), or in the frontal lobe (n = 2) and tumor grades were 2 (n = 3) or 4 (n = 2). Three of five patients were alive at the end of 5 years follow-up and progression was seen in two patients. One of these five patients withdrew from systematic epilepsy follow-up and discontinued the ASM on his own.

## Discussion

The present study analyzed retrospectively long-term seizure outcomes of patients with grade 2–4 glioma in a 5-year follow-up. Several outcome measures were used for seizure freedom, including achieved 12 month remission at some point of the disease, longest seizure-free period and seizure freedom at the end of follow-up. In a progressive disease like glioma, epilepsy outcome assesments should include measures to also identify temporary effects. The key findings are, that 1) nearly 60 % of patients were seizure-free for at least 12 months at some point during follow-up with 40 % of patients remaining seizure-free during the last year of follow-up 2) factors related to the tumor correlated with the likelihood of seizure freedom, and 3) occurrence of focal aware or focal impaired awareness seizures correlated with treatment resistance.

The characteristics of the patient population are similar to those described in other studies, apart from the slightly higher male to female ratio (1.9) in the present study, compared to the incidence ratio reported (1.4. to 1.6 [18]). In addition, we included frontotemporal tumors in the group of large/multilobar tumors accounting for a difference in tumor locations found compared to another finnish population-based cohort of 331 adults [19].

In the current patient cohort the prevalence of epilepsy in diffuse brain glioma patients was 75,6%. This is slightly higher than the 56 % prevalence reported in a large Danish population-based study [4]. Difference may be partly explained by the register-data based approach in the Danish study. In addition, some of the grade 4 gliomas are missed in this patient population, because some glioblastomas are diagnosed in the neurosurgery department after biopsy without receiving further oncological treatment.

In this study, we identified similar factors to correlate with the prognosis of both glioma and epilepsy. The strongest correlation was between glioma progression and seizure freedom. In surviving patients, progression negatively correlated both with the length of seizure-free periods and seizure freedom at the end of follow-up. This is consistent with a previous finding of an increase in seizure frequency as a sign of tumor recurrence [3]. An important observation for patient follow-up in clinical practice was that in patients first achieving a 12 month-seizure-free period, seizure recurrence coincided with glioma progression in the majority (60,7%) of the patients. Lower grade of glioma, epilepsy as first symptom, younger age and IDH mutation previously found to associate with better survival outcome of glioma correlated with longer seizure-free periods in this study [12], [13], [14]. However, in this small study population, analyzing survivors and deceased patients separately, most of these differences failed to reach statistical significance regarding seizure freedom. In patients with grade 2 or 3 glioma, epilepsy as first symptom indicated better survival than seen in patients with other presenting symptom of glioma. This is in agreement with post-resection occurrence of seizures associating with an increased mortality risk amongst glioma patients [4].

Age and tumor location have correlated with uncontrolled epileptic seizures after oncological treatment in low-grade glioma [11]. In this study, higher age correlated negatively with seizure-freedom during the last year of follow-up, but there was no statistically significant correlation between age and the longest seizure-free time period. Tumor location failed to correlate statistically significantly with seizure freedom at the end of follow-up. There was a slight trend of seizure freedom more often achieved in patients with frontal tumors, as described earlier [11].

A correlation between focal aware seizure and treatment resistance has been previously reported in low-grade glioma [20]. We found the occurrence of focal aware and focal impaired awareness seizures associated with treatment resistance compared to patients with only focal to bilateral tonic-clonic seizures. Focal aware and focal impaired awareness seizures may indeed be more refractory, as reported in other focal epilepsy populations [21], [22]. However, there may be problems in the reporting accuracy and classification for focal aware seizures and focal impaired awareness seizures beyond glioma associated symptoms.

Cancer patients with comorbidity, including glioma patients, have poorer survival than those without comorbidity [23], [24]. Comorbidities have been associated with poor seizure outcomes [15]. Somatic comorbidity correlated with shorter survival in this patient cohort and there was a trend towards worse epilepsy outcome. The use of a comorbidity index combining the number and severity of diseases might have provided additional information on the possible correlation between somatic comorbidities and the outcome of epilepsy. Psychiatric comorbidity was more common in surviving than deceased patients. Several different mechanisms may lead to glioma associated psychiatric symptoms. There can be a direct effect on brain functioning and in addition, psychological distress and mood issues may be caused by the serious illness and/or adverse effects of treatment [25]. It is possible, that patients surviving longer are more likely to have time to express, and to receive treatment for psychiatric symptoms than patients with a rapidly progressing disease.

Tumor-directed treatments are increasingly recognized as potentially effective options leading to improved seizure control. Studies in patients with glioma have suggested that when a gross total or subtotal resection is possible, most of the patients achieve a favorable Engel scale I at 1 year [7], [8]. An effect on seizure control has also been associated with radiation therapy and chemotherapy during 6–12 months follow-up [9], [10]. In this study, resection, radiotherapy, chemotherapy or chemoradiotherapy had no positive correlation with seizure freedom. Lack of effect may be due to the small study population, nor did we estimate the extent of resective surgery. Alternatively, this may be viewed as reflecting therapy effects remaining temporary in diffuse glioma patients.

In long-term survivors with grade 2 glioma, quality of life and performance status remains stable while the glioma does not progress [26]. This study confirms that in malignant glioma the prognosis of epilepsy is also more often malignant and may contribute to poor quality of life. According to this, efforts to develop more effective treatments for glioma are also efforts to improve epilepsy treatment and quality of life.

The present study has an advantage of representative, well-defined population-based data with few dropouts and long follow-up. The Helsinki population-based patient cohort includes different phases of glioma progression and treatment likely to be met in real-life neurology practice. Included were all new glioma-related epilepsy patients in Helsinki diagnosed during 2013–2015, as well as patients with more benign diffuse gliomas, still receiving treatment ten years after the diagnosis. The major limitations of the study are its retrospective nature and small patient number. Self-reported seizure counts may be erroneous as in epilepsy patients in general. Seizures with impaired awareness are associated with memory lapse of the incidence and their occurrence is often underestimated [27]. In order to reduce the uncertainty with histopathological diagnosis and oldest treatment protocols, patients with histopathological diagnosis prior to 2005 were excluded. Gliomas were classified according to histopathological analysis at time of diagnosis and were not re-classified according to WHO 2016 Classification of Tumors of the Central Nervous System. Information on IDH mutation analysis is limited and we didn’t estimate the extent of the resection causing category resection to contain both gross total resections and subtotal resections. A significant proportion of patients died during follow-up, causing shorter time periods for observation of seizure occurrence. By analyzing only grade 2 gliomas, this would have been avoidable, but the results of the present study suggests that in surviving patients the prognosis of epilepsy is similar in low (grade 2) and high (grade 3 and 4) grade diffuse gliomas. Follow-up of seizure-free time was less than five years (mean 53 months, median 58 months) for patients whose first symptom was other than epileptic seizure, so it was not possible to compare seizure-free times in patients whose first symptom was a seizure vs another symptom.

## Conclusions

In our mixed cohort of patients with glioma-related epilepsy from Helsinki, we found that it is likely to achieve a seizure-free period over one year if the glioma is not progressing rapidly. Our results support the prognosis of glioma to be the most important factor influencing the outcome of epilepsy in long term follow-up.

## Ethical statement

The study conforms to the Finnish legislation concerning medical research, and the study permission was granted by the Helsinki University Central Hospital (HUCH) Neurocenter Institutional Review Board.

## Declaration of Competing Interest

The authors declare that they have no known competing financial interests or personal relationships that could have appeared to influence the work reported in this paper.
